# To what extent do prescribing practices for hypertension in the private sector in Zimbabwe follow the national treatment guidelines? An analysis of insurance medical claims

**DOI:** 10.1186/s40545-017-0125-7

**Published:** 2017-12-04

**Authors:** Victor Basopo, Paschal N. Mujasi

**Affiliations:** International Master in Health Economics & Pharmacoeconomics, Barcelona School of Management, Universitat Pompeu Fabra, Balmes 132, 08001 Barcelona, Spain

**Keywords:** Compliance, Essential Medicines List, Hypertension, Insurance medical claims, Prescribing practices, Private sector, Zimbabwe, Standard treatment guidelines

## Abstract

**Background:**

Hypertension is the most prevalent cardiovascular disease in Zimbabwe. The prevalence of Hypertension in the country is above 30% regardless of the cut off used. Currently, majority of patients in Zimbabwe seek health care from the private sector due to limited government funding for the public health sector. However, Standard treatment guidelines for hypertension are only available in the public sector and are optional in the private sector. This study assesses compliance of private sector prescribing to Standard Treatment guidelines for hypertension.

**Methods:**

We reviewed hypertension prescription claims to a private health insurance company in Zimbabwe for the period Jan 1-Dec 31 2015. We used the last prescription claimed in the year on the assumption that it represented the patient’s current treatment. Prescription data was analyzed by comparing medicines prescribed to those recommended in the Zimbabwe 7th Essential Medicines List and Standard Treatment Guidelines 2015. We used Microsoft Excel© 2010 to conduct the analysis.

**Results:**

A total of 1019 prescriptions were reviewed. Most patients were either on mono or dual therapy (76%). The mostly prescribed class of antihypertensive as first line were Angiotensin Converting Enzyme Inhibitors /Angiotensin Receptor Blockers. Regardless of whether they were being used as first, second or third line this class of antihypertensives emerged as the most prescribed (639 times). Only 358 (35%) prescriptions were compliant with standard treatment guidelines; the rest (661) did not meet several criteria. Areas of non-compliance included use of second line medicines as first line, failure to consider patient characteristics when prescribing, use of contraindicated medicines for certain patients, clinically significant interactions among prescribed medicines and illogical combinations that predispose patients to toxicity.

**Conclusion:**

The poor compliance to standard treatment guidelines observed in our study indicates need to improve prescription practices for Hypertension in the private sector in Zimbabwe for its cost-effective management among the covered patients. However, further investigation is needed to understand the drivers of the prescribing habits and the non-compliance to the Essential Medicines List and Standard Treatment guidelines observed. This will enable design of appropriate educational, managerial and economic interventions to improve compliance.

## Background

Globally, Cardiovascular disease is the leading cause of mortality, accounting for about a third of deaths [1]. Cardiovascular disease is a group of diseases comprising endocarditis, hypertension, cardiac failure, acute pulmonary oedema, angina pectoris and acute myocardial infarction. By 2014 deaths, from cardiovascular disease were ranked fourth among the top 10 causes of mortality in those over 5 years of age in Zimbabwe [2]. Hypertension is the most prevalent cardiovascular disease in Zimbabwe [3]. The prevalence of Hypertension in the country is 30% regardless of the cut off used [4–6]. There is also a 4% prevalence of severe undiagnosed hypertension in females and 3.7% in males [7, 8]. Thus, Hypertension, whose role in cardiovascular diseases is well established, is a growing medical problem in Zimbabwe.

The Zimbabwean government recognizes the growing importance of non-communicable diseases (NCDs) including Hypertension and has prioritized their management in the national health strategy [9]. The government, through the Ministry of Health has the largest network and infrastructure in the country to support health care activities in the form of hospitals (referral, provincial, district and rural hospitals) and clinics [2]. However, there is limited government funding for the provision of the required health care including the management of NCDs. The Ministry of Health and Child Care’s 2016 budget allocation for example, was 8.3% of total government budget expenditure; this is less than the 15% agreed at the Abuja Declaration of 2000 and the Sub-Saharan average of 11.3% [10]. Given that 60.5% of government funding goes to employment costs, the basic health system in Zimbabwe is highly dependent on donor funding and individual patient payments, with the later reported to be 54.1% of total health expenditure at district hospitals by the end of 2015 [10]. Individual patient payments comprise direct payments to health care providers (out of pocket) and contributions to private health insurance or medical aid societies.

Due to the limited government funding for public sector health services in Zimbabwe, an increasing number of patients are forced to seek health care from the private sector. However, clinical practice guidelines are only available in the public sector. A multi-disciplinary team, the National Medicines Therapeutics Policy and Advisory Committee, is tasked by the Ministry of Health and Child Care to develop the Essential Medicines List and Standard Treatment Guidelines for Zimbabwe (EDLIZ) for the common diseases affecting the population. The Standard Treatment Guidelines outlined in EDLIZ are mandatory in the public sector but optional in the private sector. However, they are the only available clinical practice guidelines in the country and ideally should guide clinical practice in the private sector as well. Private health care providers tend to rely mainly on pharmaceutical company representatives as their source of prescribing information. This raises questions about the quality of care provided in the private sector, particularly whether private patients are being given the best possible care as intended by EDLIZ 2015.

### Literature review

#### Recommendations for management of hypertension

Hypertension is defined as systolic blood pressure of 140 mmHg or higher or a diastolic blood pressure of 90 mmHg or higher [11]. High blood pressure is associated with an increased risk of stroke, myocardial infarction, heart failure, renal failure and cognitive impairment [11]. The complications of hypertension are related to either sustained elevations of blood pressure, with consequent changes in the vasculature and heart, or to the accompanying atherosclerosis that is accelerated by long-standing hypertension [1, 12].

The management of Hypertension involves a combination of life style interventions and the use of therapeutic agents [3, 12–19]. The goal of hypertension treatment using therapeutic agents is to keep the blood pressure under control and to manage all the identified risk factors for cardiovascular disease, including lipid disorders, glucose intolerance or diabetes, obesity and smoking [15].

Standard treatment guidelines as outlined in EDLIZ 2015 make the following recommendations in selecting medicines for high blood pressure for adults: start with first line medicine; start with the lowest recommended dose; if ineffective or not tolerated change the medicine or add a medicine from another class [3]. The therapeutic agents recommended in EDLIZ 2015 for management of Hypertension are in line with what is being used in other parts of the world [13], as presented in the Table 1 below.

**Table 1:** Recommendations for Management of Hypertension in Zimbabwe

Categorization	Therapeutic group	Recommended medicines	Dosage and Prescribing notes
First Line	Thiazide diuretic	• Hydrochlorothiazide	hydrochlorothiazide 12.5 – 25 mg once a day. Unwanted side effects include raised plasma glucose, uric acid, and cholesterol and reduced plasma potassium
	Calcium Channel blockers	• Nifedipine • Amlodipine	Nifedipine slow release 10- 40 mg once or twice a day or Amlodipine 5-10 mg once a day.
Second line	Angiotensin converting enzyme (ACE) inhibitors	• Enalapril • Lisinopril	Enalapril 5-40 mg once a day or Lisinopril 5-40 mg once a day. Unwanted side effects are reported as a persistent cough that might occur in 10–25% of the patients, angioedema, and postural hypotension. An additional warning is that all ACE inhibitors can cause excessive hypotension and renal failure is also listed. In the event of a cough developing, Angiotensin Receptors Blockers (ARBs) can be substituted for ACE inhibitors. Hyperkalaemia can develop with the concomitant administration of ACE inhibitors with potassium supplements or potassium retaining medicines and this should only be done with careful monitoring of serum potassium.
	Angiotensin receptor blockers (ARBs)	• Losartan	Losartan 25-100 mg once or twice a day.
	Beta blockers	• Atenolol	Atenolol 50 mg once a day. Unwanted side effects include precipitation or exacerbation of asthma, heart failure, impaired glucose control, fatigue and peripheral vascular disease.
	Alpha blockers	• Prazosin • Doxazosin	Prazosin 0.5 – 5 mg twice or three times a day; or Doxazosin 4- 16 mg once a day.

Most patients will require more than one medicine to achieve control of their blood pressure [14, 16]. Patients of African origin respond well to treatment with calcium channel blockers and diuretics but have smaller blood pressure reductions with Angiotensin Converting Enzyme (ACE) inhibitors, Angiotensin Receptor Blockers (ARBs) and Beta blockers [3, 15, 16]. Beta blockers are not a preferred initial therapy for hypertension because clinical outcome benefits have not been as well established as with other agents [13, 15, 16]. Evidence linking Atenolol to higher rate of stroke among the elderly compared to other anti-hypertensives has led to its use in those over 60 years being discouraged unless there are compelling indications [15, 16].

The Zimbabwe clinical practice guidelines also provide an insight into what are considered logical combinations in the Zimbabwean context as shown in the Table 2 below.

**Table 2:**
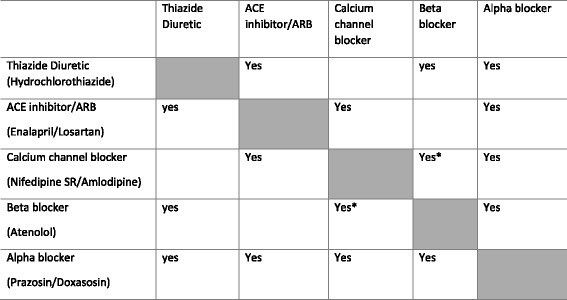
Suggested combinations of Antihypertensive Medicines for Management of Hypertension in Zimbabwe

Logical combinations ^*^ Verapamil, a calcium channel blockers and beta blockers are absolutely contraindicated. (Extract from EDLIZ 2015)

According to the EDLIZ 2015, during selection of medicines for management of Hypertension, medicine interactions should always be considered such as in cases of concurrent use of nonsteroidal anti-inflammatory drugs (NSAIDs), aminophylline, corticosteroids etc. whenever cases of resistant hypertension are encountered [3]. Angiotensin converting enzyme inhibitors and ARBs should not be used in combination but rather ARBs should be used as an alternate to ACE inhibitors in patients who develop a persistent cough [15, 16]. Thiazides and beta blockers have been shown to be an effective combination for reducing blood pressure, but since both classes can cause hyperglycemia, the combination should be used with caution in patients at risk of developing diabetes [16].

Diabetes is one of the common co-morbidities with Hypertension and can influence the choice of medicines to manage Hypertension. Hydrochlorothiazide can impair glucose tolerance exacerbating hyperglycemia in Diabetes [3]. Further, beta blockers have a potential to mask symptoms of hypoglycemia in insulin dependent diabetics [3]. The use of low dose Thiazides in diabetics, is recommended if need dictates otherwise patients should be switched to an ACE inhibitor, calcium antagonist or alpha blocker if unwanted effects appear [3].

### Rationale and significance of the study

The emergence of cardiovascular disease including Hypertension as the single most important cause of death in Zimbabwe after communicable diseases and the increasing number of patients seeking medical care in the private sector, make it necessary to study how hypertension patients are being managed in the private sector. This will help determine if patients are receiving proper care and if changes need to be made to current treatment practices.

The study uses prescription claims for hypertension medicines submitted to one medical aid society in Zimbabwe during 2015. Through an analysis of the claims data, the study compares the observed prescribing practices and medicines used with what is recommended in the 7th Essential Medicines List and Standard Treatment Guidelines for Zimbabwe, 2015 (EDLIZ 2015). The study demonstrates use of routinely available data through insurance claims to monitor adherence to guidelines and provides information which can be used to improve management of hypertension in the private sector in Zimbabwe. This would contribute to cost effective management of hypertension and reduction of the high mortality rates arising from diseases of the circulatory system. The study also adds to the body of knowledge on the management of Hypertension in Zimbabwe.

### Aim and objectives

The aim of this study was to determine, through an analysis of insurance claims data, compliance of private medical practitioners’ prescribing practices with the 7th Essential Medicines List and Standard Treatment Guidelines for Zimbabwe, 2015 (EDLIZ 2015) recommendations on the pharmacological management of hypertension.

The specific study objectives were to:

- Describe private medical practitioner’s prescription practices for hypertension, specifically the extent of use of the medicines recommended in EDLIZ 2015 for management of hypertension
- Identify instances where the private medical practitioner’s prescription practices do not comply with EDLIZ 2015 specifically existence of any clinically valid interactions between the prescribed medicines for the hypertensive patients in the study and use of combinations of medicines that are considered inappropriate per the standard treatment guidelines outlined in EDLIZ 2015

## Methods

This was a retrospective descriptive cross sectional study using secondary data. The study focused on Hypertensive patients who were covered by the Medical Aid Society of Central Africa (MASCA) and received treatment from private sector health facilities (hospitals and clinics) during the period 1 January 2015 to 31 December 2015. This included patients covered by all reimbursement schemes under the society receiving care from all levels of the health care system and from all types of prescribers. The MASCA remains one of the few viable medical insurance schemes in Zimbabwe hence the choice to study its members. MASCA has a membership of 15,000 spread across the country giving it a national character [20].

We obtained claims data submitted by patients in Zimbabwe to the Medical Aid Society of Central Africa (MASCA) during the period under study (1 January 2015 to 31 December 2015). Each claim related to a single prescription. The study was limited to medicine claims with medicines used for the management of hypertension. Thus, we extracted data on reimbursement claims containing hypertension medicines listed in EDLIZ 2015, filed with MASCA during the study period. Prescription data extracted from the MASCA database contained patient characteristics such as age, sex and race in addition to all the medicines reimbursed in the year. The information was provided in Excel format, from which hypertension prescription information was extracted manually, to create another Excel file that contained hypertension medicines only. The International Classification of Diseases, 10th Revision, Clinical Modification (ICD-10-CM) which physicians and other providers use to code all diagnoses, symptoms, and procedures recorded in hospitals and physician practices [21] is yet to be adopted so information captured by medical aids does not include diagnosis [20]. Hence, we used the prescribed medicines to identify hypertension patients. For these patients, we also collected data on other medicines for chronic conditions prescribed concurrently during the period to assess the appropriateness of combinations and clinically relevant drug interactions. We assumed that patients filed claims for all the chronic medications they acquired during the year through the scheme. The data collected represents a census of all active MASCA members and dependents suffering from hypertension who filed in claims during the 12-month study period.

The last prescription claimed by each patient during the 12-month period was used in the analysis, based on the assumption that it represented the patient’s current treatment. Collection of data covering the 12-month period made it possible to capture prescribing information from lost patients due to inability to continue making contributions, death or exhaustion of allocated benefits for the year.

We conducted a descriptive and comparative analysis of the prescription data gathered using Microsoft Excel 2010. The basis for comparison of the observed prescription practices was the 7th Essential Medicines List and Standard Treatment Guidelines for Zimbabwe 2015 (EDLIZ 2015).

## Results

### Study sample

The study sample comprised of Hypertension prescriptions claims for 1019 patients that were submitted to MASCA in the period Jan 1-December 31, 2015. Majority of the prescription claims (58%) were for Male patients and the remaining 42% were for female patients.

Patients of European and Asian origin accounted for the bulk (65%) of the prescription claims and the majority (58%) of these claims were for patients over the age of 60 (Table 3).

**Table 3:** Sample Characteristics

		Number	Percentage
Gender	Male	423	42%
	Female	596	58%
Total		1019	100%
Race	White	538	53%
	Asian	123	12%
	Black	358	35%
Total		1019	100%
Age	Below 30 Years	6	1%
	31–40 Years	40	4%
	41–50 Years	128	12%
	51–60 Years	253	25%
	Over 61 Years	592	58%
Total		1019	100%

### Prescription practices

#### Number of prescribed hypertension medicines per patient

**Fig. 1:**
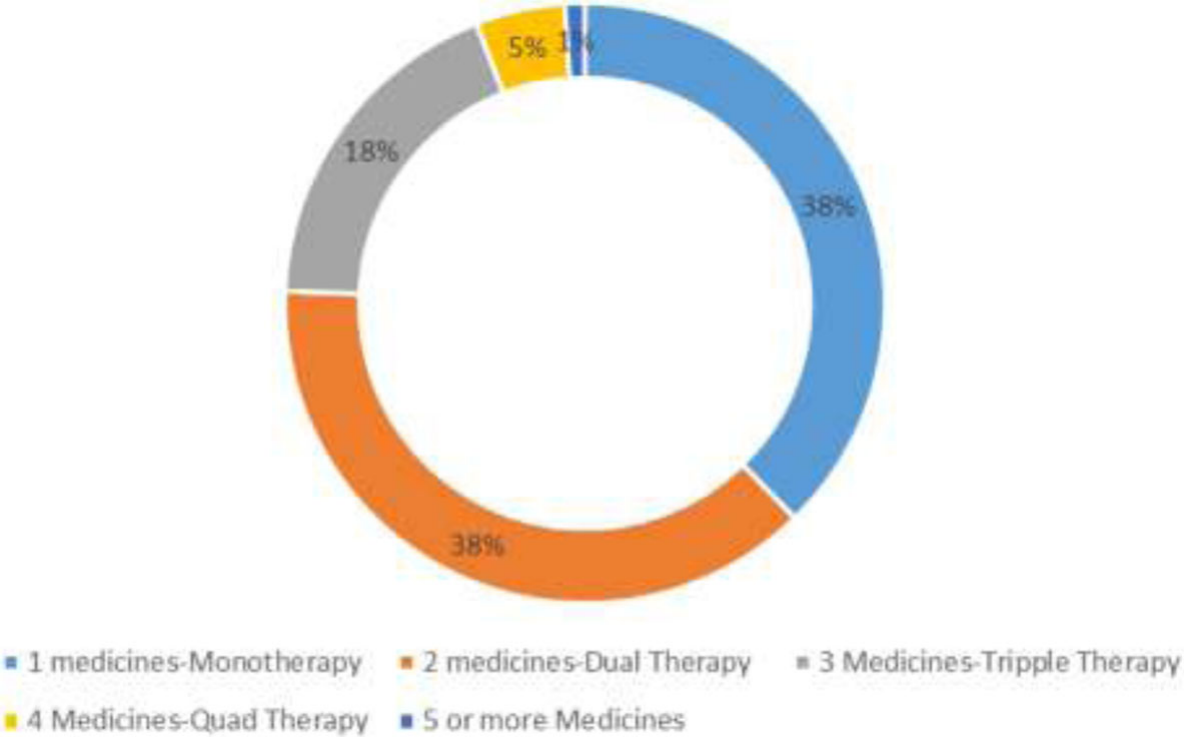
Number of Antihypertensive medicines prescribed per patient

The number of prescriptions containing one medicine; Monotherapy (384; 38%) was found to be almost equal to that with two medicines; dual therapy (387; 38%). Overall, about three quarters (76%) of patients were either on mono or dual therapy for their hypertension. The number and proportion of prescriptions with three medicines; triple therapy (188, 18%) was greater than both those on four medicines; quad therapy (49; 5%) and those with five or more medicines (11; 1%) as illustrated in Fig. 1.

#### Prescription of anti-hypertensive first line therapy by therapeutic class

For patients who were on mono therapy, we considered the prescribed medicine as their first line therapy. For those who were on two or more medicines, if any of the medicines prescribed was in the first line therapeutic class as recommended by the EDLIZ 2015 (as per Table 1) we considered that as their first line therapy. The assumption was that this was the initial medicine prescribed for them and the others having been added as required to achieve better hypertension control.

**Fig. 2:**
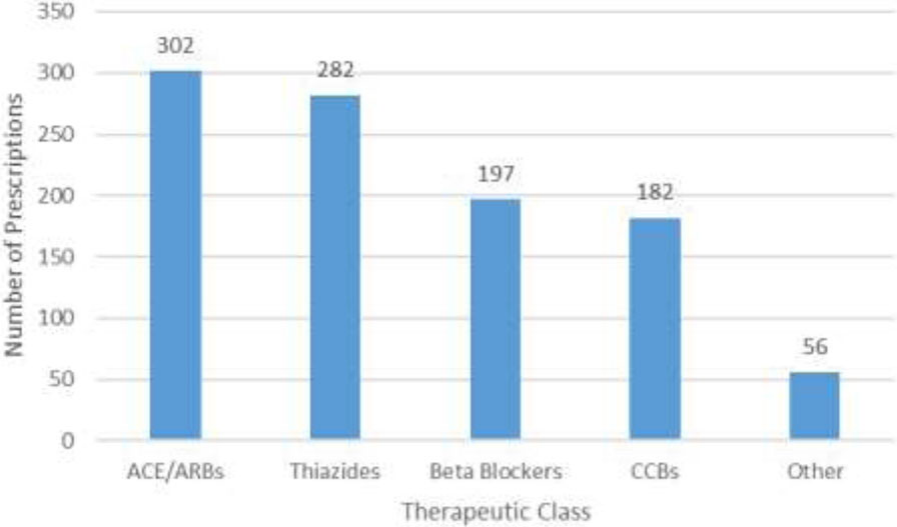
Prescriptions of first line medicines by therapeutic class. ACE/ARBs - angiotensin converting enzyme inhibitors or angiotensin receptor blockers. CCBs - calcium channel blockers. Other - other anti-hypertensives

The most prescribed medicines as first line therapy were found to be ACE inhibitors/ARBs (29.6%), closely followed by thiazides (27.7%). Beta blockers, calcium channel blockers and other medicines contributed 19.3%, 17.9% and 5.5% of the first line therapy respectively (Fig. 2).

#### Commonly prescribed antihypertensive medicines by therapeutic classes

**Fig. 3:**
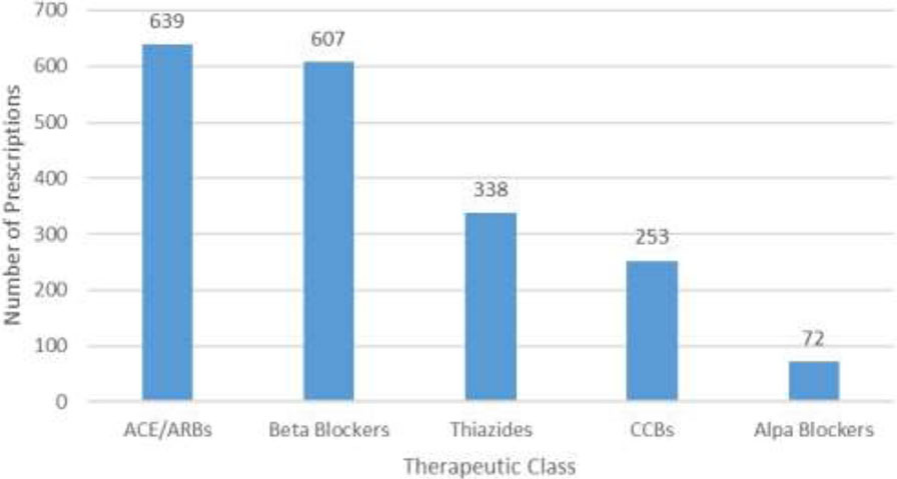
Commonly prescribed Antihypertensives. ACE/ARBs – ACE inhibitors and angiotensin receptor blockers. CCBs-calcium channel blockers

Figure 3 below shows the commonly prescribed therapeutic classes of Hypertension medicines regardless of whether they were being used as first, second or third line. Angiotensin converting enzyme inhibitors/Angiotensin Receptor Blockers emerged as the most prescribed (639 times) followed by Beta blockers (607), Thiazide diuretics (338), Calcium channel blockers (253) and Alpha blockers (72) in that order.

#### Other co-prescribed medicines

**Fig. 4:**
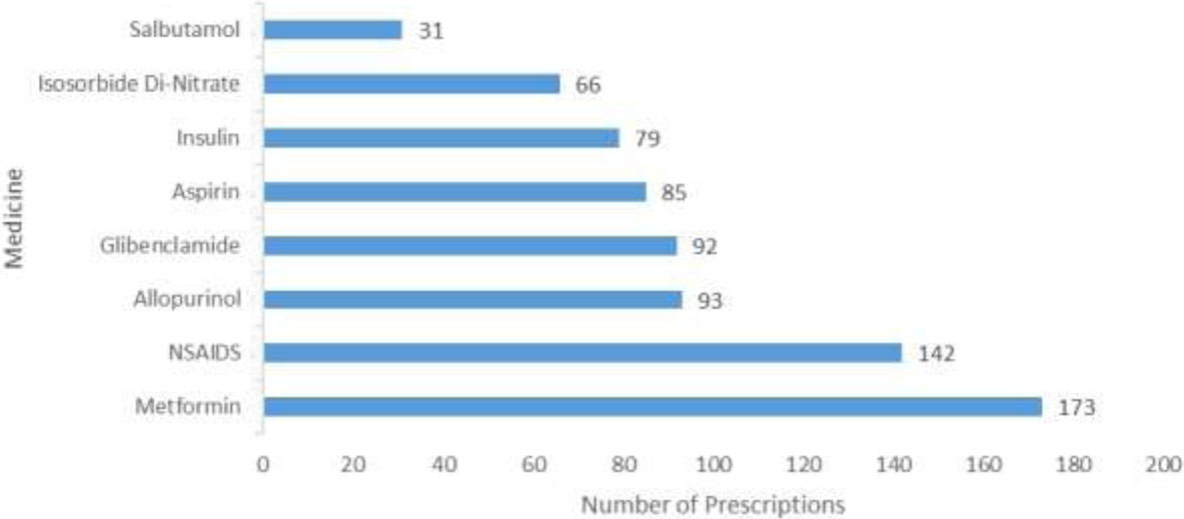
Commonly co-prescribed medicines (Non-anti hypertensives). NSAIDs-Non-Steroidal Anti Inflammatory Drugs

We reviewed the prescription data to establish the other medicines co-prescribed with Antihypertensives amongst the study sample. Figure 4 shows the other medicines that were commonly prescribed with antihypertensives. Anti-diabetic medicines were the other class of medicines most often prescribed for the hypertensive patients in our study followed by non-steroidal ant-inflammatory drugs (NSAIDs).

Metformin, Glibenclamide and Insulin are all indicated for the management of Diabetes [3]; Allopurinol for Gout; Isosorbide Dinitrate for Angina; Salbutamol for Asthma and Aspirin for the reduction of cardiovascular risk [3, 22]. Diabetes was the most common co-morbidity (173 cases), while the existence of Angina required the administration of Isosorbide Dinitrate in 66 cases. Non-steroidal anti-inflammatory drugs were found in 142 prescriptions while aspirin and allopurinol were co-prescribed in 85 and 93 prescriptions respectively.

### Prescription compliance with standard treatment guidelines

**Fig. 5:**
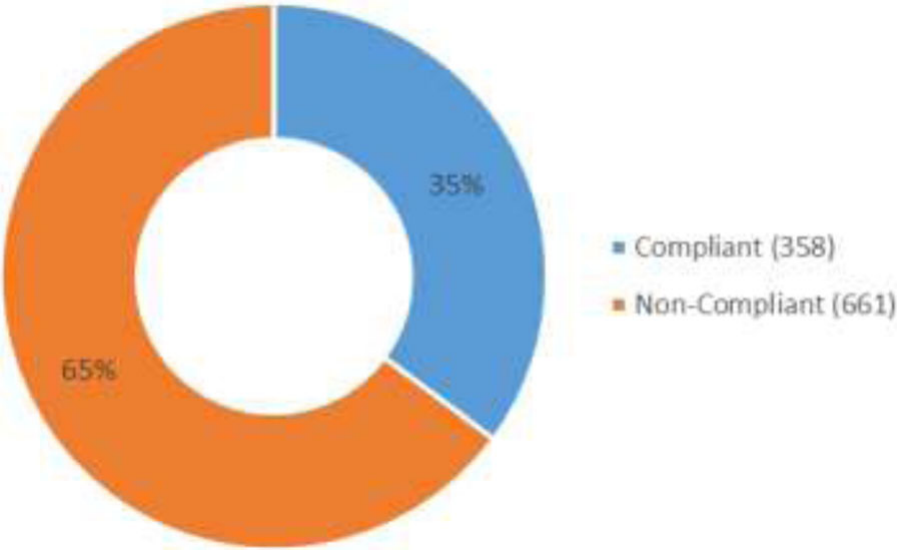
Prescription compilance with Standard Treatment Guidelines, 2015

Almost two-thirds (65%) of the prescriptions were found not to be in line with EDLIZ 2015 recommendations. Compliance was referenced to correct use of first line medicines, prescribing to the appropriate population subgroups, administering medicines in the same therapeutic class to the same patient and avoidance of known drug interactions (Fig. 5).

### Reasons for non-compliance

#### Prescription of inappropriate medicines

Half of the non-compliant cases (52%) emanated from the inappropriate use of second line medicines to initiate therapy in patients. The balance of the noncompliance was accounted for by the prescribing atenolol to patients over 60 years of age (29%), use of beta blockers or ACE inhibitors/ARBs as monotherapy in people of African origin (10%), co-prescribing medicines in the same therapeutic class (4.7%), combining potassium sparing agents together (3%) and using beta blockers and ACE inhibitors as dual therapy in patients of African origin (Table 4).

**Table 4:** Reasons for non-compliance

Compliance issue	Number of prescriptions	Percentage
Second line medicines being prescribed as first line	342	52%
Atenolol being prescribed to patients above the age of 60 years.	189	29%
Beta blocker or an ACE inhibitor being used as monotherapy in people of African origin	69	10%
Two medicines from the same therapeutic class being co-prescribed,	31	5%
ACE/ARBs inhibitors in combination with potassium sparing diuretics	19	3%
Combination of a beta blocker and an ACE inhibitor or ARB to treat people of African origin	11	2%
Total	661	100%

#### Clinically significant interactions or contraindications

Clinical interactions can result in reduced effectiveness of medicines or an increase in side effects both to the disadvantage of the patient [6, 17, 18]. Potential interactions documented in the standard treatment guidelines were identified in 406 prescriptions (about 40% of the prescriptions). Table 5 below shows the main interactions identified. The use of ineffective therapy in patients of African origin though not strictly an interaction was included here because of the impact it has on poor hypertension control.

**Table 5:** Clinically significant interactions or contraindications

Interaction or contraindication	Number of prescriptions	Percentage
NSAIDs-non-steroidal anti-inflammatory drugs	142	35%
Beta blockers or ACE/ARBs used as monotherapy in patients of African origin,	69	17%
Atenolol administered to diabetics	53	13%
Thiazide-Amiloride combination containing hydrochlorothiazide 50 mg per tablet	45	11%
Medicines from the same therapeutic class being administered together	31	8%
Potassium sparing agents and ACE/ARBs administered together	19	5%
Hydrochlorothiazide prescribed to diabetics	17	4%
Hydrochlorothiazide prescribed to patients with gout	13	3%
Beta blockers and ACE/ARBs used as dual therapy in patients of African origin.	11	3%
Atenolol prescribed to asthmatics	6	1%
Total	406	100%

## Discussion

Calcium channel blockers (CCBs) and thiazide diuretics are the recommended first line medicines for Hypertension as per the EDLIZ 2015 guidelines [3]. However, from the survey, private practitioners seem to prescribe ACE/ARBs the most followed by Thiazides. This is at variance with EDLIZ 2015 that places ACE/ARBs as second line medicines that are only to be prescribed in the event of treatment failure or in situations where patients develop intolerance [3]. It is highly unlikely that the 30% of patients on ACE/ARBs had experienced treatment failure or intolerance suggesting doctor’s preference to ACE/ARBs for initiating therapy.

Beta blockers and calcium channel blockers were used as initial therapy but to a lesser extent than thiazides and ACE/ARBs. The previous edition of EDLIZ 2015 had atenolol as a first line medicine therefore the continued use of the drug could reflect the slow pace at which doctors adjust to changes in guidelines. The same argument could be advanced for the lower than expected use of CCBs that only became first line in the current edition. Several guidelines [15–17] now discourage the routine use of beta blockers as first line because of poor health outcomes, therefore one can argue that their continued use as observed in this study disadvantages patients. Angiotensin receptor blockers and ACE inhibitors were used as first line possibly because of the influence of pharmaceutical promotion. A couple of studies suggest that promotion by pharmaceutical companies particularly through advertising contributed to adoption of newer hypertensive medicines in the United States [23–25]. It is also however possible that doctors in our study were following some international guidelines [16, 17] which recommend Angiotensin receptor blockers and ACE inhibitors as first line for white patients. As earlier indicated, about 53% of the prescriptions surveyed in our study were for white patients.

The recommended approach to the pharmacological management of hypertension is stepped care whereby if a single drug does not adequately control blood pressure, drugs with different modes of action can be combined to effectively lower blood pressure while minimizing toxicity. Rational drug prescribing in the circumstance is then defined as the use of the least number of drugs to obtain the best possible effect in the shortest period and at a reasonable cost [26]. The bulk of the patients (62%) in the study had their hypertension managed using two medicines or more, consistent with international best practice [16–18, 27]. A study of patients attending a cardiology clinic in India showed a similar trend with most of the patients on multiple therapies with two combined antihypertensive [28]. This pattern is encouraged by international guidelines which state that prescribing small doses of different classes of antihypertensive medicines is more beneficial than prescribing a high dose of one antihypertensive.

Given a sizeable proportion of severe undiagnosed hypertension (3–4%) in Zimbabwean population [5–7] it is possible that patients are presenting at health facilities late; this may explain the observation that most patients were on two or more anti-hypertensives since most guidelines recommend commencing treatment with two drugs whenever systolic blood pressure is above 160 mmHg [17, 18]. In such a scenario of severe undiagnosed hypertension in Zimbabwean population the relevance of monotherapy as observed in our study with 38% of patients being on monotherapy becomes questionable since it may not provide adequate control [25, 27]. Indeed, in a study by Al-Drabah et al. in which the majority of subjects were prescribed monotherapy, the researchers observed that target BP control was not achieved in most patients [29] which implies that monotherapy may not be sufficient for achieving adequate BP control in majority of patients [30].

Only 35% of the surveyed prescriptions were found to be following the Zimbabwe standard treatment guidelines and therefore can be considered compliant. Different studies have found different levels of compliance to treatment guidelines for hypertension in various contexts. Like this study, a study conducted in Malaysia observed that doctors poorly adhered to Malaysian Clinical Practice guidelines [30]. In another study in Malaysia as well, 85.3% of prescriptions at a Cardiac clinic were in accordance with guidelines [31]. In contrast to our study, findings from a study in Eritrea found that prescribing practice for hypertension followed the Eritrean National treatment guideline 2003 [32].

Several Studies have shown that application of guidelines to clinical practice improves treatment outcomes, especially better BP control [33–35]. Thus, the low level of adherence observed in our study is of concern. It is however important to note that guidelines are just to guide but physicians need to follow a patient-centric approach. Thus, it is not always surprising that inconsistencies exist between recommended and observed treatment approaches because clinicians sometimes individualize therapy based on specific patient characteristics and response to treatment. Several studies show that adherence to clinical guidelines and recommendations are not all uniform; they vary by time period and country, and by characteristics of patients and physicians [36–39].

We observed various areas of noncompliance in our study, ranging from failure to consider patient characteristics to using second line medicines as first line. Beta blockers and ACE/ARBs are known for being less effective as monotherapy in patients of African origin [3] yet these were prescribed to 22% of the surveyed African patients. Bearing in mind the Zimbabwean population is largely black, this is a huge concern. The use of second line medicines as first line was associated with beta blockers, ACE inhibitors or ARBs, all listed as second line in EDLIZ 2015. Possible explanations could be the effects of pharmaceutical promotion or doctors aligning themselves to guidelines that promote their use as such [15, 16, 18, 19]. Continued use of Atenolol as observed could be a sign of doctors reacting slowly to the new guidelines thus perpetuating old practices. Also, recent guidelines [13, 15, 16] discourage the use of Atenolol in elderly patients but doctors seem unaware because 32% (189 out of 592) of this age group was on Atenolol. Instances of prescribing medicines from the same therapeutic class as observed offers no advantages to the patients and instead a combination of medicines from different therapeutic classes is recommended in the event of treatment failure [3, 17, 25]. The combination of beta blockers and ACE/ARBs as observed in 3% of prescriptions has not been proven to have any synergistic hypotensive effects and is often discouraged [19].

The recommended chronology of adding antihypertensive medication is that of add-on i.e. in the event of treatment failure a new class of medicine is added onto the existing one unless there is intolerance which necessitates the withdrawal of the first medicine [3, 27]. It therefore follows that the most prescribed medicines should be the first line agents followed by the second line and so forth. It was interesting to note that the two most prescribed classes were ACE/ARBs (639) and beta blockers (607) yet these are considered second line in EDLIZ 2015. Popularity of ACE/ARBs could be two-fold namely the effect of pharmaceutical promotion or private sector doctors using international guidelines which recommend these as reference [16–18]. Ideally thiazides and CCBs should have been appearing on the bulk of the prescriptions but given that guidelines are recent, one may think that this was a transition period, with growing usage expected over time. However, thiazide-type diuretics have always been first line in previous editions perhaps indicating private sector prescribing is not aligned to EDLIZ 2015. The widespread use of atenolol observed in our study could be attributed to the previous edition that had placed it as first line and doctors were still to adjust to the new recommendations. The low usage of alpha blockers is in line with expectations as they are reserved for the resistant cases and men beyond 50 years with benign prostrate hypertrophy [3, 18].

Interactions between medicines or between medicine and patient characteristics can lead to poor hypertension control or an increase in toxicity [3, 16]. Non-steroidal anti-inflammatory drugs reverse the effects of antihypertensive medicines [3], predisposing patients to complications [12], but they were being routinely prescribed in 142 patients, about 14% of the study population. Regular use of the NSAIDs could be linked to joint problems often experienced by the elderly who constituted more than half the study population.

Insurance has been shown to increase access to antihypertensive drugs by patients [39–43]. Given the positive impact of Insurance on overall access to antihypertensive drugs, an important question is whether insurance also plays a role in shaping prescribing patterns of antihypertensive agents in line with treatment guidelines. Given the drive for efficiency and effectiveness by insurance companies, one would have expected to see a higher level of compliance to treatment guidelines in this cohort of antihypertensive patients who have private medical insurance through MASCA. This expectation is based on the popular view that health insurance companies in general are more likely to exercise management controls to encourage prescribing that is consistent with established national guidelines particularly if the recommended therapy also represents the lowest cost alternative. One possible explanation for the observed low compliance in our study despite coverage by health insurance is that the insurance society has relatively weak controls over prescribing.

Studies have shown that prescribing in the private for profit sector tends to be worse than in public sector as indicated by poorer compliance with Standard Treatment Guidelines (STGs), and lower use of Essential Medicines Lists (EML) and generic drugs [44]. However, it’s important to note that the private sector in Zimbabwe is not bound to follow EDLIZ 2015 which is only mandatory in the public sector. Indeed, in many countries in Africa, the private sector is encouraged but not obliged to prescribe from EML as may be the case for public health centers [45]. In the absence of any binding guideline or effective regulations on prescribing behavior for clinicians, the current prescription pattern observed in this cohort of MASCA clients is probably a reflection of the mixed effect of the preferences of clinicians, the hypotensive efficacies of medications, and the tolerance levels of patients.

A methodological strength of our study is that it uses readily available claims data and demonstrates use of routinely available data to evaluate and monitor prescribing habits. This can help rapid identification of the necessary modifications to prescribing habits to achieve rational and cost effective treatment. Additionally, our analysis is based on observations of actual prescribing practices as recorded by clinicians rather than on reported practices which may be subject to recall bias. Since we used data for all clients covered by MASCA which has the largest coverage for private sector clients, we can be confident that our data provides a fair representation of hypertensive prescription habits in the private sector in Zimbabwe. Few studies have studied medicines use in the private sector [44]. The number of studies in the private-for profit sector is very small which precludes accurate comparison with other settings. This study contributes to the current knowledge and incipient knowledge on prescribing patterns in the private sector.

Findings from this study point to the need to improve prescription practices for Hypertension in the private sector in Zimbabwe. However, further investigation is needed to understand the drivers of the prescribing habits and the non-conformance to the EDLIZ 2015 observed in this study. Assuming that EDLIZ 2015 provides the most appropriate guidance for hypertensive treatment in Zimbabwe, our study indicates that there is room for significantly improving the cost effectiveness of hypertensive treatment among the covered patients. We recommend that the Association of Healthcare Funders of Zimbabwe (AHFoZ) through its membership actively encourages the use to EDLIZ 2015 by all private medical practitioners or instead develops its own clinical practice guidelines in consultation with all the stakeholders involved. Medical aids societies are encouraged to provide training and regular feedback to medical practitioners, through representative associations such as the Zimbabwe Medical Association or the College of Primary Care Physicians of Zimbabwe to improve their adherence to guidelines. A low percentage of medicines prescribed from an EML may highlight private sector prescriber’s lack of knowledge on the role of the EML in cost-effectiveness optimization.

### Study limitations and suggestions for future research

We did not have access to diagnosis or detailed clinical information for the patients in our study. Thus, we used the medicines as a surrogate for the diagnosis hypertension. We could also not distinguish between newly diagnosed or long standing hypertensive patients or even determine the severity of the hypertension. This lack of diagnosis and other clinical information (e.g. comprehensive data on co-morbidities over and above the few we concentrated on) made it difficult to accurately ascertain the appropriateness of prescribing especially when combined medicines were prescribed. Collecting this detailed information may provide different insights on the appropriateness of current prescribing and its adherence to guidelines.

The study was carried out soon after the updating of the guidelines. It is not clear to what extent poor dissemination or lack of knowledge about the guidelines could be responsible for the observed prescribing practices. Identifying which factors were at play was out of scope but an interesting area for further research. What determines the choice of antihypertensive therapies is a question of vital commercial, medical and public health importance. Thus, there is need to investigate factors that explain the observed practice. Also, there is need track trends in usage given the guidelines are new and see how this changes over time. A better understanding of the causes of the observed prescribing behavior could help target interventions to improve prescribing of hypertension for the covered patients. These would ideally involve a mix of educational components, managerial components and economic interventions depending on the identified causes.

This being a cross sectional study design considering the most recent prescription claim for each patient, any earlier switch of drug treatment could not be considered in the study design. Thus, we could not examine switches among antihypertensive drugs classes either due to non-response or side effects as this may explain the observed prescription patterns. Also, we did not have data about the prescribers-age, type of provider (Specialists or not) level of care (hospital or health center) etc. which might influence the prescribing patterns of antihypertensive drugs.

It would also be interesting to study the costs of care, clinical outcomes (was hypertension being controlled) and quality of life of the patients in the study and if this was affected by the prescribing practices observed. Such research may offer stronger grounds for use of the EDLIZ 2015 by MASCA since these are not currently mandatory.

## Conclusion

The study uses prescription claims for hypertension medicines submitted to one medical aid society in Zimbabwe during 2015. It compares the observed prescribing practices and medicines used with what is recommended in the 7th Essential Medicines List and Standard Treatment Guidelines for Zimbabwe, 2015 (EDLIZ 2015). The poor compliance to standard treatment guidelines observed in our study indicates need to improve prescription practices for Hypertension in the private sector in Zimbabwe for its cost-effective management among the covered patients. However, further research is needed to understand the drivers of the prescribing habits and the non-compliance to the Essential Medicines List and Standard Treatment guidelines observed. This will enable design of appropriate educational, managerial and economic interventions to improve compliance.

## Abbreviations

ACE: Angiotensin converting enzyme
AIDS: Acquired immuno deficiency syndrome
ARB: Angiotensin receptor blockers
BP: Blood pressure
CCBs: Calcium channel blockers
EDLIZ 2015: 7th Essential medicines list and standard treatment guidelines for Zimbabwe
EML: Essential Medicines Lists
HIV: Human immunodeficiency virus
ICD-10-CM: International Classification of Diseases, 10th Revision, Clinical Modification
JNC: Joint National Committee
MASCA: Medical Aid Society of Central Africa
NCD: Non-communicable diseases
NSAIDs: Non-steroidal anti-inflammatory drugs
STGs: Standard treatment guidelines
WHO: World Health Organization
